# Intraspecific variation in *Gyrodactylus mediotorus* and *G. crysoleucas* (Gyrodactylidae) from Nearctic shiners (Leuciscidae): evidence for ongoing speciation, host-switching, and parasite translocation[Fn FN1]

**DOI:** 10.1051/parasite/2024023

**Published:** 2024-06-11

**Authors:** Chahrazed Rahmouni, Mária Seifertová, Megan G. Bean, Andrea Šimková

**Affiliations:** 1 Department of Botany and Zoology, Faculty of Science, Masaryk University Kotlářská 2 611 37 Brno Czech Republic; 2 Texas Parks and Wildlife Department 5103 Junction Highway Mountain Home TX 78058 USA

**Keywords:** Monogenea, *Gyrodactylus*, Leuciscidae, North America, Haptor, Nuclear genes

## Abstract

A parasitological investigation of *Cyprinella venusta* and *Notropis* cf. *stramineus* sampled in Texas, USA, in the Guadalupe River, revealed the presence of *Gyrodactylus crysoleucas* Mizelle and Kritsky, 1967 on *C. venusta*, and *Gyrodactylus mediotorus* King, Marcogliese, Forest, McLaughlin & Bentzen, 2013 on both fish species*.* This represents new leuscicid fish hosts and locality records for these two gyrodactylids. *Gyrodactylus crysoleucas* previously identified from both non-native Californian *Notemigonus crysoleucas* and from farmed stocks in Minnesota demonstrated intraspecific variability in terms of morphology and genetics as a local adaptation associated with isolation by distance. Results further confirmed *G. crysoleucas* as alien in the western USA and suggested host-switching involving *C. venusta* and *N. crysoleucas*. Conservative morphology and genetics on the part of *G. mediotorus* from *C. venusta* and *N.* cf. *stramineus* (Guadalupe River) was observed, while higher genetic divergence in the ITS sequences associated with morphological discrepancy was found between the studied *G. mediotorus* specimens and those of *Notropis hudsonius* than when considering the parasites of *Notropis texanus*. The separation of *G. mediotorus* into geographical subgroups may indicate ongoing speciation linked to the Pleistocene glaciations in North America, and to hydrographic barriers that facilitated separate evolutionary paths leading to speciation. We suggest that deep investigations of *Gyrodactylus* populations will help to understand the speciation of these parasites and their adaptation to Nearctic fish hosts.

## Abstract (fr)

Une enquête parasitologique sur *Cyprinella venusta* et *Notropis* cf. *stramineus* échantillonnés au Texas, États-Unis, dans la rivière Guadalupe, a révélé la présence de *Gyrodactylus crysoleucas* Mizelle et Kritsky, 1967 sur *C. venusta*, et de *Gyrodactylus mediotorus* King, Marcogliese, Forest, McLaughlin & Bentzen, 2013 sur les deux espèces de poissons. Ceci représente de nouveaux poissons Leuciscidae hôtes et des nouvelles localités pour ces deux Gyrodactylidae. *Gyrodactylus crysoleucas*, identifié précédemment à partir de *Notemigonus crysoleucas* californiens non indigènes et de stocks d’élevage du Minnesota a démontré une variabilité intraspécifique en termes de morphologie et de génétique en tant qu’adaptation locale associée à l’isolement par la distance. Les résultats ont en outre confirmé que *G. crysoleucas* était exotique dans l’ouest des États-Unis et ont suggéré un changement d’hôte impliquant *C. venusta* et *N. crysoleucas*. Une morphologie et une génétique conservatrices pour *G. mediotorus* de *C. venusta* et *N.* cf. *stramineus* (rivière Guadalupe) ont été observées, tandis qu’une divergence génétique plus élevée dans les séquences ITS, associée à une divergence morphologique, a été trouvée chez les spécimens étudiés de *G. mediotorus* et de *Notropis hudsonius* lorsque l’on considère les parasites de *Notropis texanus*. La séparation de *G. mediotorus* en sous-groupes géographiques peut indiquer une spéciation continue liée aux glaciations du Pléistocène en Amérique du Nord et à des barrières hydrographiques qui ont facilité des chemins évolutifs séparés menant à la spéciation. Nous suggérons que des études approfondies sur les populations de *Gyrodactylus* aideront à comprendre la spéciation de ces parasites et leur adaptation aux poissons hôtes néarctiques.

## Introduction

The taxonomic identification of parasitic organisms is an essential task in biodiversity assessment, restoration ecology, and conservation biology, and is crucial for a better understanding of host biogeography and parasite evolution. Generally speaking, nominal species are distinct morphological and genetic clusters separated from other taxa by speciation or separately evolving metapopulation lineages [80, 94]. The evolutionary processes in monogeneans, a highly host-specific ectoparasitic group [85, 105], are known to be driven by new host colonization, ecological adaptation, and host speciation [46, 106, 111]. However, the taxonomy of monogeneans is challenging due to the occurrence of cryptic species characterized by only a few distinguishing traits. Such species, however, exhibit divergent genetic characteristics. The integration of DNA sequences into classification has enabled the recognition of more cryptic monogenean species [7, 34, 83].

Cryptic diversity in monogeneans was previously detected using nuclear markers in gill-specific *Dactylogyrus* spp. (Dactylogyridae) from native northwest African *Luciobarbus* spp. (Cyprinidae) [83] despite the generally known presence of relevant morphological characters to discriminate among *Dactylogyrus* spp. The genus *Gyrodactylus* von Nordmann, 1832 (Gyrodactylidae) is certainly more taxonomically challenging than *Dactylogyrus* and its systematics represents a demanding task [58, 78]. As one of the most diverse and widespread taxa within Monogenea [1, 2, 15, 36, 78], *Gyrodactylus* spp. are known for their highly conservative morphology, making the use of molecular data essential for accurate identification [42, 60, 81, 82]. In contrast to *Dactylogyrus*, the morphology of each of the sclerotized marginal hooks, anchors (hamuli), transverse bars, and the male copulatory organ (MCO), alone, can be insufficient for recognising a species of *Gyrodactylus*. Thus, a pairwise comparison of the DNA sequences of various nuclear genes, mainly those of the most informative internal transcribed spacer (ITS rDNA), is required [75,113]. Cryptic *Gyrodactylus* lineages were reported in several freshwater systems on the basis of sequences of the ITS rDNA, including, for instance, *Gyrodactylus rugiensis* Gläser, 1974 and *Gyrodactylus rugiensoides* Huyse and Volckaert, 2002 from European gobiids with a genetic divergence varying between 1.5% and 1.8% [42], and the widespread *Gyrodactylus cichlidarum* Paperna, 1968 and *Gyrodactylus ulinganisus* García-Vásquez, Hansen, Christison, Bron and Shinn, 2011, both parasitizing African cichlids and a range of other teleost hosts worldwide, with a genetic divergence reaching 5.2% [31]. Distinct genetic markers like the cytochrome oxidase subunit II (COII) revealed up to 17.2% intraspecific genetic divergence in the case of *Gyrodactylus bullatarudis* Turnbull, 1956, *Gyrodactylus poeciliae* Harris and Cable, 2000 and *Gyrodactylus turnbulli* Harris, 1986, all known to parasitize South American and Caribbean *Poecilia* guppies [107]. Moreover, cryptic diversity in *Gyrodactylus* was also observed in non-native (introduced) fish populations of European catfishes [73]. In the Nearctic region, Rahmouni *et al.* [82] identified complex speciation and diversification processes in *Gyrodactylus* communities of native Leuciscidae (Cypriniformes) and hypothesized recent/ongoing gene flow. In this case, however, *Gyrodactylus huyseae* Rahmouni, Seifertová and Šimková, 2023 parasitized two evolutionarily closely-related leuciscid hosts: the striped and spottail shiners, *Luxilus chrysocephalus* Rafinesque, 1820 and *Notropis hudsonius* (Clinton, 1824), respectively (the latter species being previously a member of *Luxilus* (Mayden 1989)), both potentially occurring in an overlapped distributional range [74]. Most recently, the Nearctic *Gyrodactylus mediotorus* King, Marcogliese, Forest, McLaughlin and Bentzen, 2013, a species originally described from the spottail shiner, *N. hudsonius*, from Canada, was identified on the weed shiner, *Notropis texanus* (Girard, 1856), in Wisconsin – however, with only a slight genetic divergence [56]. Molecular phylogeny has revealed host-switching in *Gyrodactylus*, which is now considered a common speciation scenario in these parasites due to hyperviviparity and a monoxenous life cycle involving transmission by fertile adults [5, 9]. Host-switching scenarios in *Gyrodactylus* were thought to be less likely due to the phylogenetic dissimilarity between native and new (exotic) fish hosts [27]. Hypothetically, once a gyrodactylid has colonized a suitable natural host population, one would expect a high probability of speciation. From an evolutionary perspective, such sympatric speciation can occur even on phylogenetically distant hosts [6]. While sympatric speciation by host-switch on closely related hosts was evidenced in *Gyrodactylus* spp. parasitizing European marine gobiids [41, 42], speciation by geographic isolation (allopatric speciation), host-switch, and instant isolation by host specificity were all revealed in *Gyrodactylus* from European salmonids [63]. At a larger scale, Boeger *et al.* [3] emphasized the importance of adaptive radiation and nonvicariant processes in the historical diversification of viviparous gyrodactylids. With respect to the Nearctic region, Rahmouni *et al.* [82], using both 18S rDNA and ITS markers and morphological features of the attachment organ (haptor), reported potential host-switching for the generalist *Gyrodactylus hanseni* Rahmouni, Seifertová and Šimková, 2023 found on unrelated hosts: *L. chrysocephalus* and the creek chub *Semotilus atromaculatus* (Mitchill, 1818). In their study [82], *Gyrodactylus colemanensis* Mizelle and Kritsky, 1967 was reported for the first time on a wild-living leuciscid, the cutlip minnow *Exoglossum maxillingua* (Lesueur, 1817), whereas all previous records mostly involved non-native salmonids introduced to North America mainly for farming purposes [12, 14, 18, 33, 65, 104, 109]. To a lesser degree, Nearctic *Gyrodactylus* that have been introduced to Europe have been shown to accidently infect native freshwater fish [72]. Further, the ornamental (aquarium) fish trade in exotic hosts is one of the main pathways for the co-introduction of non-native monogeneans, leading to parasite spillover towards native fish fauna. This type of invasive scenario was observed in exotic poeciliid fishes (Cyprinodontiformes) native to southeast Asia [47] and to the neotropics [43, 53].

During our recent survey of fish parasites in Texas, we studied a set of monogeneans collected from two shiner species naturally occurring in southcentral USA watersheds, the blacktail and sand shiners, *Cyprinella venusta* Girard, 1856 and *Notropis* cf. *stramineus* (Cope, 1865), respectively. Nearctic minnows of the shiner clade represent one of the most taxonomically complex clades of Cypriniformes due to conserved morphologies in numerous taxa [96]. Taxonomically, shiners and related minnows have been classified as members of the subfamily Pogonichthyinae since the taxonomic revision by Schönhuth *et al.* [93] and Tan and Armbruster [100], who elevated subfamilies to family rank in cyprinoids. *Notropis* and many taxa formally placed in this genus have long had an ambiguous status and this genus was considered a taxonomic repository for small, silvery shiners of uncertain placement (see [30]). Still, over 320 nominal species of Nearctic members of *Notropis* are listed in the Catalog of Fishes [29]. *Cyprinella*, like *Luxilus*, *Lythrurus*, and *Pteronotropis*, were considered subgenera within *Notropis* until Mayden [61] elevated them into different genera and relocated species. *Cyprinella venusta* Girard, 1856 is a small freshwater shiner native to the USA with a wide distributional range across the country [30, 74]. The species has also been introduced into the Pecos River near Pandale, Texas [39]. The blacktail shiner prefers flowing waters, creeks, and rocky pools and runs, and areas with little vegetation and gravelly bottoms [30]. Likewise, *Notropis stramineus* (Cope, 1865) is a small shiner native to the central part of the USA, southern Canada, and Mexico [74]. In the USA, it is found sporadically on the Edward Plateau, in the Big Bend region of the Rio Grande, and along the Red River [39], and inhabits sand and gravel runs, pools of creeks, and small to large watersheds [30].

From a species-richness perspective, parasitological investigations of *Notropis* spp. and *Cyprinella* spp., including all previous fish members of these genera, have revealed a total of 14 *Gyrodactylus* spp. [48, 49, 56, 62, 82, 86–89]. While most of the known *Gyrodactylus* spp. parasitizing representatives of the genera *Cyprinella*, *Luxilus*, *Lythrurus*, and *Notropis* exhibit strict host specificity by infecting a single host species, there are three *Gyrodactylus* spp. with more than a single recorded host. They are *Gyrodactylus baeacanthus* Wellborn and Rogers, 1967 from *C. venusta* [103] and from the comely shiner *Notropis amoenus* (Abbott, 1874) [52], *Gyrodactylus lythruri* Rogers, 1975 from the blacktip shiner *Lythrurus bellus* (Snelson, 1972), and from the pretty shiner congener *Lythrurus bellus* (Hay, 1881) [88], and finally *G. mediotorus* found on *N. hudsonius* and *N. texanus* [48, 56]. Furthermore, *Gyrodactylus atratuli* Putz and Hoffman, 1963, known from a range of leuciscid dace species of the genera *Margariscus* and *Rhinichthys* [35, 79, 82], was also found to parasitize the spotfin shiner *Cyprinella spiloptera* (Cope, 1867) [49].

The goal of the present study was first to perform a morphological and genetic study of *Gyrodactylus* specimens collected from *C. venusta* and *N. stramineus* in Texas. Previously, *Gyrodactylus* spp. had never been documented for *N. stramineus*. We investigated the degree of similarity/dissimilarity to known *Gyrodactylus* spp. according to relevant morphological characters of the haptor, mainly, and genetic markers. Further, we summarized the data on the host specificity of the *Gyrodactylus* spp. studied and tried to infer their historical transmissions in the Nearctic region.

## Material and methods

### Ethics and permits

Fish were collected under Texas Parks and Wildlife’s state employee biologist permit for Megan Bean.

### Shiner fish sampling and genetic identification

A total of 34 shiner specimens of *C. venusta* and 14 specimens *N. stramineus* were collected in Texas (USA) on June 5, 2023, in West Mud Creek, Neches River, and on May 30, 2023, in the Guadalupe River (30.073490, −90.140196), near Kerrville, Texas, respectively. Fish specimens were identified *in situ* by local co-workers (listed in acknowledgements). We further performed molecular analysis on the samples and obtained sequence data from the partial cytochrome *b* (cyt-*b*) mitochondrial gene to confirm the identity of the investigated shiners. Mitochondrial DNA of host species was isolated from fin clips preserved in 96% ethanol using a DNeasy^®^ Blood and Tissue Kit (QIAGEN, Hilden, Germany), following the manufacturer’s instructions. The Cyt-*b* gene was amplified using the forward primer GluF (5′-AACCACCGTTGTATTCAACTACAA-3′) and reverse primer ThrR (5′-ACCTCCGATCTTCGGATTACAAGACCG-3′) [57]. PCR reactions consisted of 1 U of Taq DNA polymerase (Thermo Fisher Scientific, Waltham, MA, USA), 1 × PCR buffer, 1.5 mM MgCl_2_, 0.4 mM of each dNTP, 0.4 μM of each primer, and an aliquot of 30 ng (1 μL) of genomic DNA in a total volume of 25 μL. PCR was carried out in a Mastercycler ep gradient S (Eppendorf AG, Hamburg, Germany) with the following steps: 2 min at 94 °C followed by 39 cycles of 45 s at 92 °C, 90 s at 48 °C, and 105 s at 72 °C, and 7 min of final elongation at 72 °C. The PCR product was purified by EPPiC Fast (A&A Biotechnology, Gdynia, Poland) and was sequenced directly in both directions using the same primers as in the amplification reaction. The initial amplification was carried out using a BigDye^®^ Terminator v3.1 Cycle Sequencing Kit (Applied Biosystems by Thermo Fisher Scientific) and an Applied Biosystems 3130 Genetic Analyzer (Applied Biosystems). Raw nucleotide sequences were edited using Sequencher software v. 5.0 (Gene Codes, Ann Arbor, MI, USA) and aligned using ClustalW [101] as implemented in MEGA v. 11 [99]. The identification of shiners based on a sequence similarity approach was carried out using the Basic Local Alignment Search Tool (https://blast.ncbi.nlm.nih.gov/Blast.cgi: blastn, default settings). Newly generated sequences for the cypriniform species were deposited in GenBank under the accession numbers **(**accession numbers indicated in Table 1**)**. In this study, shiner fish host nomenclature follows Froese and Pauly [30]. Below, we refer to the sand shiner as *N*. cf. *stramineus* (Guadalupe River) as the species is part of a species complex in southcentral USA currently being described (Kevin W. Conway, unpublished data).

**Table 1:** List of gyrodactylid species included in the phylogenetic analyses performed based on sequences of the ITS region. Monogenean species are grouped by host species and family, and geographical locality. Species used to root the phylogenetic tree are marked by “*”. Newly generated sequences are indicated in bold. Fish host nomenclature follows Fricke *et al.* [29] and FishBase [30].

Gyrodactylid species	Host species	Host Family	Locality	ITS regions
*Gyrodactyloides bychowskii	*Salmo salar*	Salmonidae	Scotland	AJ249348
*Gyrodactylus atratuli*	*Rhinichthys cataractae*	Leuciscidae	Canada	OR270008
*Gyrodactylus colemanensis*	*Exoglossum maxillingua*	Leuciscidae	USA	OR270009
*Gyrodactylus colemanensis*	*Salvelinus fontinalis*	Salmonidae	Canada	JF836142
***Gyrodactylus crysoleucas***	***Cyprinella venusta***	**Leuciscidae**	**USA**	**PP309998**
*Gyrodactylus crysoleucas*	*Notemigonus crysoleucas*	Leuciscidae	USA	KT149287
*Gyrodactylus hamdii*	*Catostomus commersonii*	Catostomidae	USA	OR269996
*Gyrodactylus hanseni*	*Luxilus chrysocephalus*	Leuciscidae	USA	OR269998
*Gyrodactylus laevisoides*	*Phoxinus eos*	Leuciscidae	Canada	KF263527
*Gyrodactylus lummei*	*Campostoma spadiceum*	Leuciscidae	USA	OR270003
***Gyrodactylus mediotorus* ‘C. venusta, *N. stramineus*’**	***Cyprinella venusta Notropis stramineus***	**Leuciscidae**	**USA**	**PP309999**
*Gyrodactylus mediotorus* ‘*N. hudsonius*’	*Notropis hudsonius*	Leuciscidae	Canada	KF178301
*Gyrodactylus mediotorus* ‘*Notropis texanus*’	*Notropis texanus*	Leuciscidae	USA	MW666182
*Gyrodactylus mendeli*	*Nocomis biguttatus*	Leuciscidae	USA	OR270004
*Gyrodactylus prikrylovae*	*Pimephales promelas*	Leuciscidae	USA	OR270005
*Gyrodactylus scholzi*	*Pimephales promelas*	Leuciscidae	USA	OR270007
*Gyrodactylus* sp. 1 ‘*Misgurnus anguillicaudatus*’	*Misgurnus anguillicaudatus*	Cobitidae	USA	MH667465
*Gyrodactylus* sp. 2 ‘*Misgurnus anguillicaudatus*’	*Misgurnus anguillicaudatus*	Cobitidae	USA	MH667466
*Gyrodactylus* sp. ‘*R. osculus*’	*Rhinichthys osculus*	Leuciscidae	USA	AY099508
*Gyrodactylus* sp. ‘*H. nuchalis*’	*Hybognathus nuchalis*	Leuciscidae	USA	OR270019
*Gyrodactylus* sp. ‘*N. crysoleucas*’	*Notemigonus crysoleucas*	Leuciscidae	USA	KT149288
*Gyrodactylus* sp. 1 ‘*R. atratulus*’	*Rhinichthys atratulus*	Leuciscidae	USA	OR270020
*Gyrodactylus* sp. 1 ‘*C. spadiceum*’	*Campostoma spadiceum*	Leuciscidae	USA	OR270016
*Gyrodactylus* sp. 2 ‘*R. atratulus*’	*Rhinichthys atratulus*	Leuciscidae	USA	OR270021
*Gyrodactylus* sp. 2 ‘*C. spadiceum*’	*Campostoma spadiceum*	Leuciscidae	USA	OR270017
*Gyrodactylus spathulatus*	*Catostomus commersonii*	Catostomidae	USA	OR270011
*Gyrodactylus stunkardi*	*Rhinichthys cataractae*	Leuciscidae	USA	OR270012
*Gyrodactylus ticuchi*	*Notropis moralesi*	Leuciscidae	Mexico	MT879676
*Gyrodactylus tobala*	*Notropis imeldae*	Leuciscidae	Mexico	MT879671
*Gyrodactylus variabilis*	*Notemigonus crysoleucas*	Leuciscidae	Canada	OR270014
*Gyrodactylus wardi*	*Catostomus catostomus*	Catostomidae	Canada	OR270015
*Ieredactylus rivuli	*Rivulus hartii*	Rivulidae	Trinidad	HQ738514
*Laminiscus gussevi	*Mallotus villosus*	Osmeridae	Iceland	HF548678

### Collection and morphological characterization of *Gyrodactylus*

Once at the laboratory, the external body surfaces, fins, and gills of cyprinid hosts were checked for the presence of gyrodactylid parasites using an MST130 stereoscopic microscope. When present, parasite specimens were removed using surgical needles and mounted on slides with a mixture of glycerine and ammonium picrate (GAP) [59]. Monogenean specimens were studied morphologically considering a total of 23 morphological characters, of which four, seven, and one corresponded to the hamuli (also termed anchors), and the ventral and dorsal bars, respectively, and eight and two characters corresponded to the marginal hooks and male copulatory organ (MCO), respectively. The terminology for the haptoral sclerites and the method of measurements follow those of Malmberg [58], Pugachev *et al.* [78], and Kritsky and Thatcher [50]. *Gyrodactylus* species were identified using original descriptions available thus far (see the result sections for references). Measurements (given in micrometers as the mean followed by the range and the number of measurements (*n*) in parentheses) and photographs were taken using an Olympus BX51 phase-contrast microscope and Olympus Stream Image Analysis v. 1.9.3 software (Olympus, Tokyo, Japan). Drawings of the haptoral sclerotized parts were made on flattened specimens using an Olympus BX51 microscope equipped with a drawing tube and edited with a graphic tablet compatible with Adobe Illustrator CS6 v. 16.0.0 and Adobe Photoshop v. 13.0 (Adobe Systems Inc., San Jose, CA, USA). Prevalence and intensity of infection were calculated according to Bush *et al.* [8]. Voucher material was deposited in the Helminthological Collection of the Institute of Parasitology, the Biology Centre of the Academy of Sciences of the Czech Republic, České Budějovice (IPCAS) under the accession numbers (see Results section).

### DNA amplification, sequencing, and phylogeny of *Gyrodactylus* spp.

*Gyrodactylus* specimens were subjected to DNA amplification and sequencing. Specimens stored in 96% ethanol were dried using an Eppendorf 5301 Concentrator. Total genomic DNA was extracted using the DNeasy^®^ Blood and Tissue Kit (QIAGEN) following the protocol for the purification of total DNA from animal tissues. Two nuclear ribosomal DNA markers suitable for the differentiation of *Gyrodactylus* spp. were used (for instance, see [10,33,60,75,113]). A fragment spanning ITS1, 5.8S and ITS2 (ITS regions) was amplified using forward primer ITS1F (5′-GTTTCCGTAGGTGAACCT-3′) [90], complementary to the sequence at the 3′ end of the 18S rRNA gene, and reverse primer ITS2 (5′-TCCTCCGCTTAGTGATA- 3′) [17], complementary to the sequence at the 5′ end of the 28S rRNA gene [17]. A partial fragment of 18S rDNA containing the V4 region, which exhibits intraspecific variation in *Gyrodactylus* [16, 60], was amplified using the primer pairs PBS18SF (5′-CGCGCAACTTACCCACTCTC-3′) and PBS18SR (5′-ATTCCATGCAAGACTTTTCAGGC-3′) [13]. Polymerase chain reactions (PCRs) for the 18S rDNA gene and ITS region were performed in a final volume of 30 μL, containing 1xPCR buffer, 1.5 mM MgCl_2_, 200 μM of each dNTP, 0.5 μM of each primer, 1 U of Taq DNA Polymerase (Thermo Scientific) and 5 μL of template DNA. The PCRs were carried out in the Mastercycler ep gradient S (Eppendorf) using the following steps: i) ITS regions: an initial denaturation at 96 °C for 3 min, followed by 39 cycles of denaturation at 95 °C for 50 s, annealing at 52 °C for 50 s, and an extension at 72 °C for 50 s, and a final elongation at 72 °C for 7 min; and ii) 18S region: an initial denaturation at 95 °C for 3 min, followed by 39 cycles of denaturation at 94 °C for 1 min, annealing at 54 °C for 45 s, and an extension at 72 °C for 1 min 30 s, and a final elongation at 72 °C for 7 min. PCR products were electrophoresed on 1.5% agarose gels stained with Good View (SBS Genetech, Bratislava, Slovakia) and then purified using EPPiC Fast (A&A Biotechnology, Gdynia, Poland), following the manufacturer’s protocol. The purified PCR products were sequenced directly in both directions using the PCR primers. Sanger sequencing was carried out using a BigDye^®^ Terminator v3.1 Cycle Sequencing Kit (Applied Biosystems) and an Applied Biosystems 3130 Genetic Analyzer. Newly-generated DNA sequences were assembled and edited using Sequencher software v. 5.0 (Gene Codes, Ann Arbor, MI, USA) and aligned using ClustalW [101] as implemented in MEGA v. 11 [99]. Sequences were further checked by the nBLAST Search Tool (https://blast.ncbi.nlm.nih.gov/Blast.cgi: blastn, default settings, access date: 11/09/2023) to assess any similarity to available congeners, then deposited in GenBank under accession numbers indicated in the Results section. Genetic divergences were estimated using uncorrected *p*-distances in MEGA v. 11 [99].

A phylogenetic tree was reconstructed based on the newly generated ITS sequences of *G. crysoleucas* and *G. mediotorus*, together with 31 DNA sequences representing 29 *Gyrodactylus* spp. retrieved from the GenBank database. The dataset included *Gyrodactyloides bychowskii* Albova, 1948, *Ieredactylus rivuli* Schelkle *et al.*, 2011, and *Laminiscus gussevi* (Bychowsky and Polyansky, 1953) Pálsson and Beverley-Burton, 1983 as the outgroup following Rahmouni *et al.* [81] (Table 1). Nucleotide sequences were aligned by multiple alignments using MAFFT v7.505 [45] and trimmed using *trimAl* v1.2rev57 [11] through plugins installed in PhyloSuite v1.2.3 [108, 110]. The plugin of ModelFinder v2.2.0 [44] in PhyloSuite v1.2.3 [108, 110] was used to determine the best-fit substitution model for the dataset. A final alignment of 33 ITS sequences composed of 927 bp was used to infer the phylogenetic relationships using Maximum Likelihood (ML) and Bayesian Inference (BI). ModelFinder v2.2.0 [44] indicated GTR+F+G4 as the best-fitting evolutionary models for ML analysis based on the corrected Akaike Information Criterion (AICc) [40, 97]. ML trees were inferred using IQ-TREE v1.5.5 [69] based on the selected model employing a sub-tree pruning and re-grafting (SPR) branch-swapping algorithm. The branch support (bootstrap support, BS) was estimated using ultrafast bootstrap approximation [64] with 1,000 replicates. BI analysis was performed using MrBayes v3.2.1 [91] and applying the GTR+I+G evolutionary model with two independent Markov Chain Monte Carlo (MCMC) simulations (six chains, 10^6^ generations, sampling frequency 100, 25% burn-in to obtain the consensus tree and posterior probability values (PP)). Chain stationarity and parameter convergence were assessed in TRACER v1.7.1 [84], with effective samples sizes (ESS) always >200 for all parameters, and via the average standard deviation of split frequencies (always well below 0.01), and post burn-in trees were summarized in a 50% majority rule consensus tree. The resulting ML and BI trees were visualized in FigTree v1.4.4 (http://tree.bio.ed.ac.uk) and manually edited on Photoshop v13.0.

## Results

Morphological characterization considering the haptoral hard parts indicated *Gyrodactylus* specimens studied herein to be highly reminiscent of already known species. Based on this statement together with DNA sequences, we confirmed the presence of *G. crysoleucas* Mizelle and Kritsky, 1967, restricted so far to either non-native [65] or captive golden shiner, *Notemigonus crysoleucas* (Mitchill, 1817) [55], and the well-known *G. mediotorus* of *N. hudsonius* [48] and *N. texanus* [56].

### Morphological characterization

Gyrodactylidae Cobbold, 1864

*Gyrodactylus* Nordmann, 1832

#### *Gyrodactylus crysoleucas* Mizelle and Kritsky, 1967 (Figures 1A–1B)

Previous records: golden shiner, *N. crysoleucas*, Rooney Pond, 17 miles east of Sacramento, California, USA [65]; captive *N. crysoleucas*, private baitfish, Minnesota [55], both in the USA.

**Figure 1:**
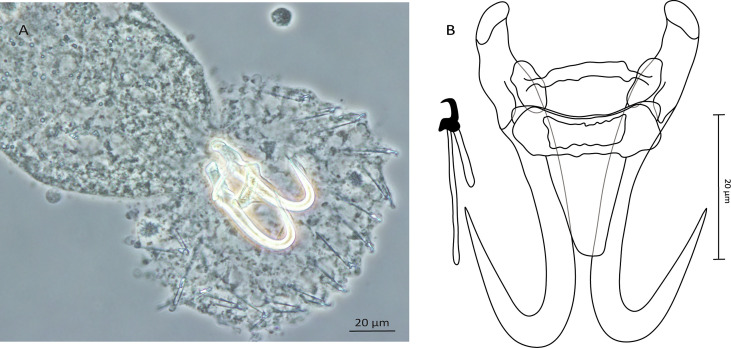
Photomicrographs (A) and line drawings (B) of *Gyrodactylus crysoleucas* from the blacktail shiner, *Cyprinella venusta*.

Present study: blacktail shiner, *C. venusta*, West Mud Creek, Neches River, Texas, USA

Site of infection: fins

Prevalence and intensity of infection: 2.9%, a single infected host out of 34 investigated, two *Gyrodactylus* specimen on a single infected host.

Voucher: IPCAS M-795

Host GenBank accession number: cyt-*b*: PP314044–PP314045

Parasite GenBank accession numbers: 18S rDNA: PP309996; ITS: PP309998

Morphology: Haptor subcircular, anchor base with folds, tips curved inward, total length 51.6 (51.2–51.9; *n* = 2); shaft slightly bowed, length 39.6 (39–40.3; *n* = 2); point curved and elongate, length 22.9 (22.5–23.4; *n* = 2); root relatively short, tapered, length 14.3 (13.1–15.5; *n* = 2). Ventral bar with short, blunt lateral processes extending out of bar, total length 23.1 (23–23.2; *n* = 2), total width 22.9 (22.7–23.1; *n* = 2), lateral processes length 2 (1.5–2.5; *n* = 2), distance between tips 22.4 (21.5–23.3; *n* = 2), median width 5.3 (5.2–5.3; *n* = 2), membrane (shield) almost trapezoid tapering posteriorly and extending almost 1/2 of length of anchor shaft, no striations or ridges were observed, length 15 (13.8–16.2; *n* = 2), width at the insertion 12.8 (12.3–13.4; *n* = 4). Dorsal bar straight with projections near each end, attenuated ends inserted into terminal plates, total length 21.9 (19.4–24.4; *n* = 2), width at midpoint 2.5 (2.2–2.7; *n* = 2). Marginal hooks total length 25.6 (25.1–26.1; *n* = 2); sickle foot noticeable with downward globose heel, prominent triangular toe, conspicuous shelf; sickle proper almost as thick as toe base, shaft length 5.2 (4.9–5.5; *n* = 2); sickle length to shaft attachment 3.5 (3.4–3.5; *n* = 2); sickle proximal width 2.9 (2.6–3.2; *n* = 2); sickle distal width 3.7 (3.6–3.8; *n* = 2)_;_ point relatively thin and slightly curved, length 1.5 (1.3–1.8; *n* = 2); filament loop extending about 1/2 of handle length, length 8.9 (8.3–9.5; *n* = 2); handle length 20.8 (19.9–21.6; *n* = 2). MCO not found.

Considering the original study by Mizelle and Kritsky [65], our specimens presented relatively (i) shorter anchors (51.2–51.9 μm in this study *vs*. 55–61 μm in the original description), and (ii) a shorter ventral bar (23–23.2 μm in this study *vs*. 25–32 μm in the original description). The shape of the marginal hooks was similar with a downward heel and prominent finger-like toe and shelf, but with a slightly thinner shaft of the sickle proper in the case of our specimens. Photographs published by Leis *et al.* [55] provided more details about the morphology of the haptoral sclerotized structures of *G. crysoleucas* and indicated that our specimens parasitizing *C. venusta* sampled in its natural southcentral range and those found on cultured *N. crysoleucas* in Minnesota were of a similar shape, and that measurements of the hard parts mostly overlapped.

#### *Gyrodactylus mediotorus* King, Marcogliese, Forest, McLaughlin & Bentzen, 2013 (Figures 2A–2D)

Previous records: spottail shiner, *N. hudsonius* (type-host), Îles de la Paix, St. Louis Lake (type-locality) and Îles Vert, St. Lawrence Lake, both in Quebec, Canada [48]; weed shiner, *N. texanus*, Lake Onalaska, Upper Mississippi River, Wisconsin, USA [56].

**Figure 2:**
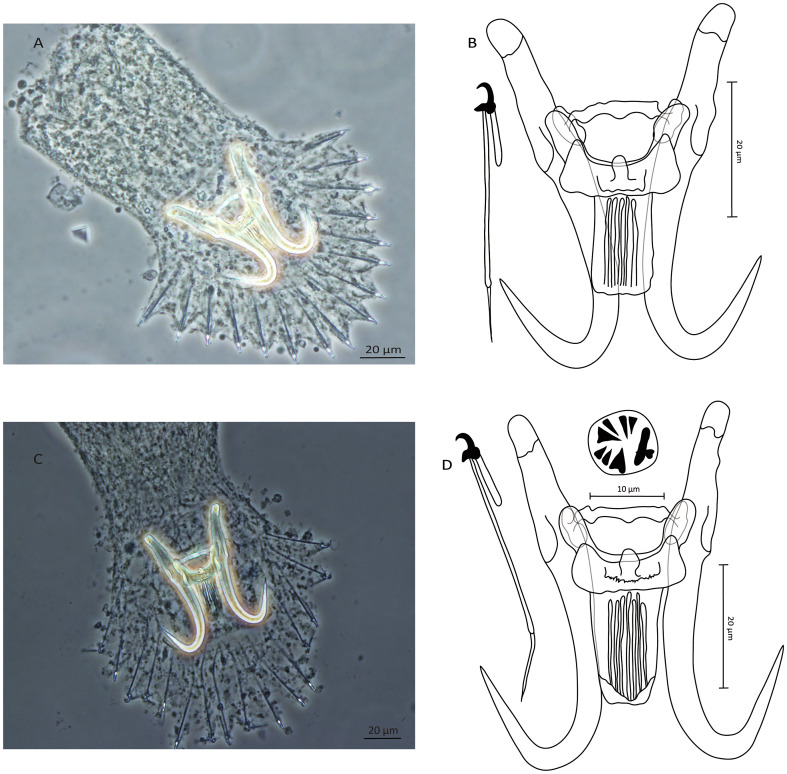
Photomicrographs (A–C) and line drawings (B–D) of *Gyrodactylus mediotorus* King, Marcogliese, Forest, McLaughlin and Bentzen, 2013 from the blacktail shiner, *Cyprinella venusta* (A–B) and from the sand shiner, *Notropis* cf. *stramineus* (Guadalupe River) (C–D).

Present study: blacktail shiner, *C. venusta*, West Mud Creek, Neches River (Figure 2A–2B), and sand shiner, *N.* cf. *stramineus* (Guadalupe River)*,* Guadalupe River (Figure 2C–2D), both in Texas, USA.

Site of infection: fins

Prevalence and intensity of infection: for *C. venusta*, 5.9%, two infected hosts out of 34 investigated, a single *Gyrodactylus* specimen on each infected host. For *N.* cf. *stramineus* (Guadalupe River), 14.3%, two infected hosts out of 14 investigated, from 1 to 3 *Gyrodactylus* specimen per infected host.

Voucher: IPCAS M-794/1-2

Host GenBank accession numbers: cyt-*b*: PP314044–PP314045 for *C. venusta*, PP314046 for *N. stramineus*

Parasite GenBank accession numbers: 18S rDNA: PP309997; ITS: PP309999

Morphology: Haptor subcircular, anchor base with folds, tips curved outward, total length 59.4 (55.9–62.6; *n* = 8); shaft slightly bowed, length 42.9 (40–45; *n* = 8); point curved and elongate, length 21.5 (20.3–22.6; *n* = 8); root long, length 20.3 (18.3–21.6; *n* = 8). Ventral bar with prominent blunt lateral processes extending out of bar, total length 33.5 (30.7–36.9; *n* = 8), total width 22.4 (20.7–24.8; *n* = 8), lateral processes length 7.8 (5.5–9.5; *n* = 8), distance between tips 26.1 (23–29.5; *n* = 8), median part with a noticeable knob, width 5.5 (4.3–6.9; *n* = 8), membrane (shield) rectangular with fine longitudinal striations, extending almost 1/2 of length of anchor shaft, length 18.1 (15.6–20.4; *n* = 8), width at the insertion 15.3 (12.3–17.6; *n* = 8). Dorsal bar straight with projections near each end, attenuated ends inserted into terminal plates, total length 23.7 (21–26.2; *n* = 8), width at midpoint 2.6 (1.8–2.9; *n* = 8). Marginal hooks total length 35.6 (31–38.8; *n* = 8); sickle foot moderate with downward globose heel, prominent triangular straightforward toe, conspicuous shelf; sickle proper almost as thick as toe base, shaft length 4.9 (4.4–5.4; *n* = 8); sickle length to shaft attachment 3.2 (2.8–3.7; *n* = 8); sickle proximal width 3.1 (2.6–3.5; *n* = 8); sickle distal width 3.4 (2.8–4; *n* = 8)_;_ point relatively thin and slightly curved, length 1.3 (1.1–1.7; *n* = 8); filament loop extending about 1/3 of handle length, length 8.5 (7.3–9.7; *n* = 8); handle ending in a noticeable posterior filament, length 30.1 (26.2–34; *n* = 8). MCO observed in a single *Gyrodactylus* specimen from *N. stramineus* cf. (Guadalupe River) with a single apical prominent spine and a single row of seven spinelets.

Size and shape of the sclerotized structures of *G. mediotorus* specimens from southeast populations of each of *C. venusta* and *N.* cf. *stramineus* (Guadalupe River) overlapped. Compared to the type-material [48], noticeable intraspecific variability was observed, mainly in terms of the (i) shorter hamuli (55.9–62.6 μm in this study *vs*. 65.7–69.7 μm in the original description), and (ii) the shorter ventral bar (30.7–36.9 μm in this study *vs*. 36.4–41.3 μm in the original description). Although well visible on the photographs, King *et al.* [48] did not mention the presence of a knob in the median part of the ventral bar or a prominent filament attachment posteriorly to the handle of the marginal hooks. The median knob of the ventral bar was later emphasized by Leis *et al.* [56] when reporting a variant of *G. mediotorus* on *N. texanus*, whereas the additional filament marking the posterior end of the marginal hooks was not highlighted. The specimens of *G. mediotorus* studied herein can be compared to the so-far-unknown *Gyrodactylus* sp. *“C. venusta”* collected recently in Mississippi [82] in having a similar haptoral morphotype, but mainly because of the presence of the ventral bar knob and the filament of the marginal hooks. Yet, considerable variation in the size of the ventral bar is observed (30.7–36.9 μm in this study *vs*. 20.1 μm in [82]). Likewise, our specimens of *G. mediotorus* possessed a longer ventral bar than *G. mediotorus* from *N. texanus* (30.7–36.9 μm in this study *vs*. 22 μm in [56]).

### Genetics and phylogenetic reconstruction

#### Gyrodactylus crysoleucas

**Figure 3:**
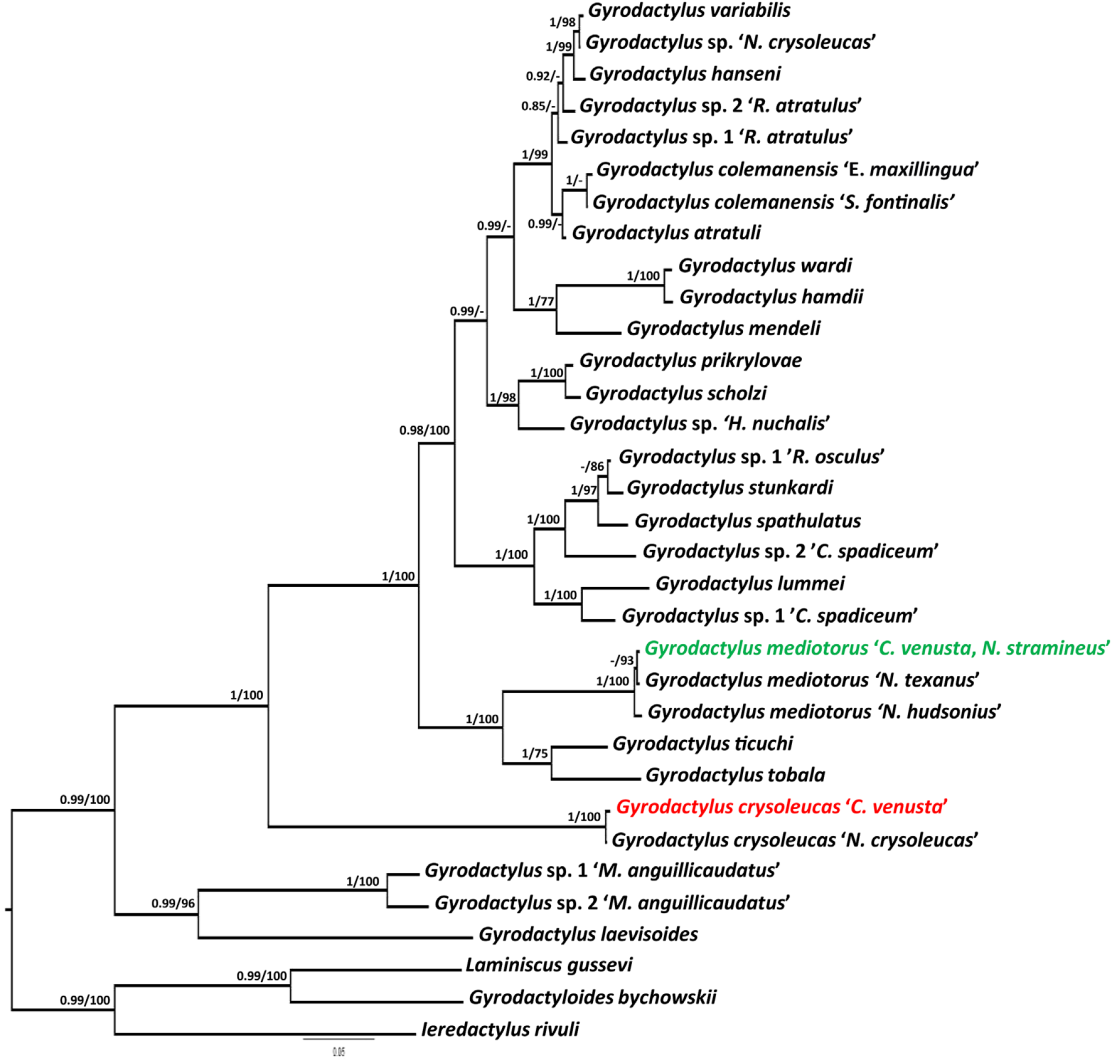
Bayesian inference (BI) phylogram of *Gyrodactylus* spp. parasitizing Nearctic Cypriniformes based on sequences of the ITS regions (927 bp). Values above branches indicate posterior probabilities (PP) from BI analyses and bootstrap support (BS) from ML. Values below 0.80 (BI) and 70 (ML) are shown as dashes.

Two fragments, the first covering the 18S rDNA (442 bp) and the second covering the ITS regions (1,207 bp), were successfully sequenced for two gyrodactylid specimens from Texas, and the newly obtained sequences were found to be identical. nBLAST search indicated *G. crysoleucas* KT149283 [55] from the farmed golden shiner *N. crysoleucas* in Minnesota (USA) as an identical match to our specimens according to sequences of 18S rDNA (100% similarity, 100% coverage), while *G. crysoleucas* KT149287 [55] was recovered as the closest match to the specimens studied herein according to the sequences of the ITS regions (99.17% similarity, 99% coverage, *p*-distance = 0.3%; 3 bp). In accordance with the morphological identification, our specimens were genetically assigned to G. crysoleucas following the delimitation by Ziȩtara and Lumme [112] and the recent findings of Rahmouni et al. [82]. Sequences of 18S rDNA further indicated *Gyrodactylus salmonis* (Yin and Sproston, 1948) from the non-native rainbow trout *Oncorhynchus mykiss* (Walbaum, 1792) from Veracruz (Mexico) (JN230350,[92]) and Washington (USA) (JF836097, [33]) as the closest match to our *G. crysoleucas* specimens from Texas (for both *G. salmonis* genetic variants; 99.55% similarity, 100% coverage, *p*-distance = 0.5%; 2 bp). Phylogenetically, *G. crysoleucas* parasitizing herein *C. venusta* from Texas together with its congener parasitizing *N. crysoleucas* from Minnesota formed a highly supported basal clade (PP = 1, BS = 100) in relation to a large clade of Nearctic *Gyrodactylus* spp. (Figure 3).

#### Gyrodactylus mediotorus

Two fragments, the first covering the 18S rDNA region (436 bp) and the second covering the ITS region (1,010 bp), were successfully sequenced for two gyrodactylid specimens from each of *C. venusta* and *N.* cf. *stramineus* (Guadalupe River) from Texas. For each gene, no intraspecific variability associated with respective fish hosts was found. Based on sequences of the 18S rDNA region, nBLAST search recovered two variants of *G. mediotorus* from the farmed spottail shiner *N. hudsonius* from Quebec (Canada) (KF178302, [48]) and the native weed shiner *N. texanus* from the Upper Mississippi in Wisconsin (USA) (MW666777, [56]) as identical matches to our specimens (respectively, 100% similarity, 100% coverage; 100% similarity, 94% coverage). Likewise, nBLAST search using the ITS sequences indicated the same variants of *G. mediotorus* from the USA (MW666182, [56]) and Canada (KF178301, [48]) as the closest matches to our specimens (respectively, 98.91% similarity, 99% coverage; 98.42% similarity, 99% coverage). In accordance with the morphological characterization, the parasite specimens we collected were genetically recognized as *G. mediotorus* following the delimitation by Ziȩtara and Lumme [116]. Yet, genetic intraspecific variation was found when comparing newly obtained and published sequences of *G. mediotorus*. *P*-distances using sequences of the ITS regions approached the limit value (1%) [116] with our specimens of *G. mediotorus* from *C. venusta* and *N.* cf. *stramineus* (Guadalupe River) from the southcentral basin (Texas) genetically closer to those found on *N. texanus* from the Upper Mississippi (Wisconsin, USA) (*p*-distance = 0.2%; 2 bp) than to *Gyrodactylus* specimens parasitizing *N. hudsonius* from the northeastern locality (Canada) (*p*-distance = 0.9%; 9 bp). With high support values (PP = 1, BS = 100), the phylogenetic reconstruction (Figure 3) indicated the monophyly of three variants of *G. mediotorus* with sister position to the clade of *Gyrodactylus ticuchi* Pinacho-Pinacho et al., 2021 (MT879676) and *Gyrodactylus tobala* Pinacho-Pinacho et al., 2021 (MT879671) parasitizing *Notropis moralesi* de Buen, 1955 and *Notropis imeldae* Cortés, 1968, respectively, from Mexico [75].

## Discussion

The present survey focused on *Gyrodactylus* spp. hosted by two Nearctic leuciscid shiners, *C. venusta* and *N.* cf. *stramineus* (Guadalupe River) occurring in their native watersheds in Texas [30, 74] Morphological study based on taxonomically important haptoral features combined with DNA sequences of the 18S rDNA and ITS regions revealed the presence of two already-known species – namely, *G. crysoleucas* and *G. mediotorus*. We recovered the former species from *C. venusta* (Figures 1A and 1B), while *G. mediotorus* was found to parasitize both *C. venusta* (Figures 2A and 2B) and *N.* cf. *stramineus* (Guadalupe River) (Figures 2C and 2D).

### *Gyrodactylus crysoleucas*: a successfully introduced parasite in the western USA

Considering the morphometric data provided by Mizelle and Kritsky [65] when describing *G. crysoleucas* from non-native *N. crysoleucas*, intraspecific variability was found in which specimens collected herein from the southcentral location exhibited shorter haptoral parts, mainly the hamuli and ventral bar. Genetically, while published and newly obtained sequences of the 18S rDNA regions of *G. crysoleucas* were conserved, sequences of the ITS regions demonstrated weak intraspecific variability on the geographical scale. Although genetic data on *G. crysoleucas* in western freshwaters are missing, the morphological variability between our specimens and the types could be explained by phenotypic plasticity and/or local adaptation in the newly invaded host (habitat) or possibly allopatric isolation. In the Nearctic region, variability in haptoral morphology across distant localities was already documented in *G. atratuli* Putz and Hoffman, 1963, a species parasitizing a wide range of leuciscid fish hosts [35, 49, 79, 82]. nBLAST search recovered *G. crysoleucas* and *G. salmonis* from non-native salmonids with highly conservative 18S rDNA sequences, which is in accordance with the morphotype of their haptoral parts, marked by poorly developed lateral processes of the ventral bar, a short posteriorly-tapering membrane, and marginal hooks with a downward heel and finger-like toe with a prominent shelf [92]. Conservative 18S rDNA sequences were reported in the recently described *G. hanseni* Rahmouni, Seifertová and Šimková, 2023 parasitizing both the striped shiner *Luxilus chrysocephalus* Rafinesque, 1820, and the creek chub, *Semotilus atromaculatus* (Mitchill, 1818), and in other *Gyrodactylus* species from native leuciscids and cultured salmonids [33, 82].

The present study adds two shiner species to the known hosts of *G. crysoleucas* across the USA; *N. crysoleucas* from California [65] and Minnesota [55], and *C. venusta* studied herein from Texas. This study presents, thus, new host and locality records for *G. crysoleucas*. To understand the geographical range of distribution of *G. crysoleucas*, it is necessary to track the historical origin of *N. crysoleucas* and *C. venusta* in the collected areas. According to the USGS database [71], both *N. crysoleucas* and *C. venusta* are naturally present in Texas and Minnesota, but not in the western part of the USA, including California. In contrast, *N. crysoleucas* was previously (late 1890s) distributed to multiple water bodies in California as a major bait and forage fish species by the California Fish Commission [26] and, since then, it has quickly spread in the western USA [98]. This is similar to the red shiner *Cyprinella lutrensis* (Baird and Girard, 1853), native to central North America west of the Mississippi River drainage [30, 74], which was successfully introduced into California’s inland waters [67]. According to Moyle [67], the golden shiner, *N. crysoleucas*, was introduced to California from more eastern watersheds, which makes *G. crysoleucas* first described by Mizelle and Kritsky [65] from *N. crysoleucas* sampled in California an alien parasite in the western part of the USA, co-introduced with golden shiner hosts. This scenario is supported by the fact that *G. crysoleucas* was found herein on wild *C. venusta* native to Texas and previously on cultured golden shiners in the far North in Minnesota [55], where they are often harvested from wild sources, which makes the possibility that *G. crysoleucas* is of western origin less likely. Nevertheless, this statement requires further investigation since the native parasite fauna in freshwater fish is still underexplored in this region. From native *C. lutrensis* in Midwestern USA (Nebraska), a single species, *G. callawayensis* Mayes, 1977, was described [62]. This was interestingly reminiscent of *G. crysoleucas* regarding the morphotype of the ventral bar characterized by poorly developed lateral processes and a short, posteriorly tapering membrane. The twisted anterior part (tips) to the hamuli is present in *G. callawayensis* [62] but not in *G. crysoleucas* which discriminates these two species. Since *C. lutrensis* is non-native in western inland waters, it would be worthwhile to investigate whether the red shiner has co-introduced its native gyrodactylids to Californian freshwaters.

Only a single parasite species, namely, *G. baeacanthus*, was formally described from *C. venusta* [103], whereas Rahmouni *et al.* [82] recently reported the presence of an undescribed species, *Gyrodactylus* sp. “*C. venusta*”, highly reminiscent of *G. mediotorus* isolated herein from *C. venusta* but also from *N.* cf. *stramineus* (Guadalupe River) (see below). In contrast, two species were recognized on *N. crysoleucas* additionally to *G. crysoleucas* [55, 65]; they are *G. rachelae* Price and McMahon, 1967 from the southeast (Tennessee) [76], and *G. variabilis* Mizelle and Kritsky, 1967 from western [65], northeast, and southcentral USA, as well as from northeast Canada [82]. Interestingly, previous [103] and current records of gyrodactylids from *C. venusta* were made from southern populations occurring in Georgia, Alabama, and Louisiana watersheds and in Texas, all representing the native distributional range of *C. venusta* [30, 70, 74]. In this study, morphological and genetic characterizations indicated that *C. venusta* hosted *G. crysoleucas* rather than *G. baeacanthus*. Two hypotheses may explain this pattern. The first one is that southcentral *C. venusta* is a native host of *G. crysoleucas*, making further widescale parasitological investigations of shiner hosts necessary to identify the gyrodactylid fauna in Texas. The second hypothesis is that *G. crysoleucas* is native to *N. crysoleucas* and infected a non-congeneric host, *C. venusta* in this case, by host-switching in overlapped habitats. Host-switching scenarios are common in *Gyrodactylus* as one major mechanism of speciation [72, 111] and have been documented in Nearctic freshwaters [82]. Further sampling of *N. crysoleucas* for the investigation of *Gyrodactylus* in Texas as well as across the whole range of its current distribution would provide more support for this hypothesis. Further, the natural host-switching of *Gyrodactylus* is known to be favorable under conditions of high parasite abundance and population growth [66] or continuous transmission ability [4]. More specific information on the infection rates of *G. crysoleucas* and on the population density of *C. venusta* would provide support for the host-switching scenario in southcentral USA.

### *Gyrodactylus mediotorus* illustrating ongoing speciation in the Nearctic

In the present study, *G. mediotorus* was isolated from the blacktail and sand shiners, *C. venusta* and *N.* cf. *stramineus* (Guadalupe River), respectively, both collected in their natural distribution range in Texas. *Gyrodactylus mediotorus* was originally described on the spottail shiner, *N. hudsonius* in Canada [48], and recently identified from the weed shiner, *N. texanus*, from the Upper Mississippi River in Wisconsin [56]. Therefore, this study presents two new shiner hosts for *G. mediotorus* and a new locality in the southeast USA. No morphological intraspecific variability of the haptoral hard parts was found in *G. mediotorus* across the studied shiner hosts. Inversely, the sclerotized structures of *G. mediotorus* collected from Texas in this study were shorter compared to those in the type-material [48]. Moreover, *G. mediotorus* appeared to possess typical features previously overlooked – specifically, the knob in the median part of the ventral bar and the prominent filament attachment posterior to the handle of marginal hooks. The filament of the marginal hooks is already known to be present in *G. spathulatus* Mueller, 1936 restricted to catostomid hosts so far [20–24, 37, 54, 68, 77, 82, 102], and in the generalist *G. stunkardi* Kritsky and Mizelle, 1968 infecting a range of Nearctic cypriniforms [21, 22, 51, 52, 68, 82, 102]. It was also reported in the new but unidentified *Gyrodactylus* spp. from the blacknose dace, *Rhinichthis atratulus*, and *C. venusta* [82]. Although morphologically similar to each other and occurring in the southcentral part of the USA, the *G. mediotorus* we studied herein and *Gyrodactylus* sp. *“C. venusta”* collected previously in Mississippi most likely belong to two distinct species due to the considerable size variation in the ventral bar [82]. DNA sequences of the ITS regions will certainly clarify the taxonomic status of *Gyrodactylus* sp. *“C. venusta”* in the future. Furthermore, our specimens and those of *G. mediotorus* from *N. texanus* [56]presented intraspecific variability. This morphological discrepancy can be related to a specific host and/or geographical locality or phenotypic plasticity as previously evidenced in *Gyrodactylus* communities [19, 72, 82]. Sequences of the 18S rDNA and ITS regions of *G. mediotorus* were successfully obtained in this study and were fully conserved at the host species level. This could be linked to the common evolutionary history of shiners in the Nearctic region [96]. On the one hand, in terms of host specificity and similar to remarks by Šimková *et al.* [95], *G. mediotorus* appears to be an intermediate specialist parasitizing congeneric as well as phylogenetically closely related non-congeneric shiner hosts across the Nearctic region. Alternatively, the presence of *G. mediotorus* on southcentral populations of *C. venusta* and *N.* cf. stramineus (Guadalupe River) could simply be an inheritance from a common ancestor or has resulted from host-switching. The former scenario seems plausible given the evolutionary relatedness between these shiners [96]. The overall *Notropis* host range associated with *G. mediotorus* and its phylogenetically closely related *G. ticuchi* and *G. tobala* may indicate that host-switching occurred from *Notropis* spp. to *C. venusta* rather than the opposite pattern (from *C. venusta* to *Notropis* spp.). Host-switching of *Gyrodactylus* also seems possible given the close phylogenetic relationship between *C. venusta* and *N.* cf. *stramineus* (Guadalupe River) and their occurrence in overlapping ecological niches in Texas. In monogeneans of the genus *Lamellodiscus* (Diplectanidae) parasitizing sympatric marine sparid fish hosts, for instance, host-parasite associations have been shown to be mostly driven by ecological factors that considerably facilitated host-switching processes [25].

In accordance with the morphological variability observed among all currently available *G. mediotorus*, *i.e.,* specimens from Canadian *N. hudsonius* (type-material), from *N. texanus* of the Upper Mississippi River in Wisconsin, and from both *C. venusta* and *N.* cf. *stramineus* (Guadalupe River) from Texas, genetic divergence was found, with *G. mediotorus* from Wisconsin and Texas (both USA) being genetically closer to each other than to the Canadian variant. Values of *p-*distances (0.9%) even approached the upper limit value (1%) of intraspecific genetic variation in the ITS regions usually considered to discriminate among *Gyrodactylus* spp. [41, 112]. Rahmouni *et al.* [82] questioned the cryptic status of *G. huyseae* found to parasitize two historically-connected hosts, *L. chrysocephalus* and *N. hudsonius,* occurring in overlapping ranges, when the genetic variation in the ITS sequences slightly exceeded the limit value and a single mutation was found in the 18S rDNA sequences. The morphological and genetic divergence of *G. mediotorus* on the geographical scale evidenced in this study may be explained by the evolutionary history of shiners – particularly, their evolution during the Pleistocene glaciations, which considerably shaped the current distribution of freshwater biotas in North America [28, 38]. Hydrographic barriers favoring separate evolutionary pathways in Nearctic freshwaters could also have been involved in creating the morphological and genetic patterns observed in *G. mediotorus* populations. Ongoing speciation in *G. mediotorus* is, thus, most likely given the complex combinations of dispersal and vicariance events that shiner hosts have experienced. The geological, climatic, and biotic factors and circumstances that have promoted such speciation remain unknown, but deeper investigations involving powerful genetic approaches using various markers, ideally both nuclear and mitochondrial, will certainly help illuminate how gyrodactylid communities are evolving and adapting to distinct Nearctic fish hosts.
